# Body weight variability and cancer incidence in men aged 40 years and older-Korean National Insurance Service Cohort

**DOI:** 10.1038/s41598-021-91601-9

**Published:** 2021-06-09

**Authors:** Yu Jin Cho, Jin Seul Kawk, Hyung-Jin Yoon, Minseon Park

**Affiliations:** 1 Department of Family Medicine, Seoul National University College of Medicine, Seoul National University Hospital, 101, Daehak-ro Jongno-gu, Seoul, 03080 Korea; 2 Department of Biomedical Engineering, Seoul National University College of Medicine, Seoul, Korea

**Keywords:** Medical research, Oncology, Risk factors

## Abstract

Repeated weight fluctuation has been proposed as a potential risk factor for increasing morbidity and mortality including cancer. We aimed to investigate the association between body weight variability (BWV) and all cancer and site-specific cancer incidence and the impact of smoking on these associations. A total of 1,759,848 cancer-free male subjects who had their weight measured at least 5 times from the National Health Insurance Service-Health Screening Cohort from 2002 to 2011 were included and followed up until 2015. BWV was defined as the average absolute difference between successive values (ASV). The risk of cancer and site-specific cancer from BWV was identified using Cox proportional hazards regression analysis using hazard ratios (HRs) and 95% confidence intervals (CIs) adjusted for potential confounders including weight, and stratified analysis was also conducted according to smoking status. During the 7,015,413 person-years of follow-up, 11,494 patients (0.65%) developed new-onset cancers. BWV was associated with a higher risk of all cancers after adjustment for confounders. The highest BWV quintile group compared to the lowest had greater risks of all cancers and site-specific cancers including lung, liver, and prostate cancer (HR 1.22, 95% CI 1.15–1.30; HR 1.22, 95% CI 1.07–1.39; HR 1.46, 95% CI 1.19–1.81; HR 1.36, 95% CI 1.15–1.62, in all cancers, lung, liver and prostate cancer, respectively). Due to small number of cancer occurrence, the risk of kidney cancer was increased, but statistically insignificant (HR 1.38, 95% CI 0.91–2.10). Similar results were observed in noncurrent smokers. However, in current smokers, the risks of all cancers and only prostate cancer were significantly increased in the highest BWV quintile group (HR 1.19, 95% CI 1.09–1.31; HR 1.51, 95% CI 1.08–2.11). The risk of kidney cancer also increased in this group, although the finding was not statistically significant (HR 1.77, 95% CI 0.87–3.63) This study suggested BWV is an independent risk factor for cancer in men, especially in lung, liver, and prostate cancer, but evidence was weaker in kidney cancer. This association remained significant only in prostate cancer in current smokers.

## Introduction

Obesity has known as a risk factor for hypertension, diabetes, cardiovascular disease, and cancer^1–4^. Intentional weight loss might be an effective strategy to prevent and reduce the risk of the progression of some chronic diseases. However, there is still debates as to whether weight loss eventually leads to prevention of long-term consequences of noncommunicable diseases such as cardiovascular disease and cancer^4,5^.

Moreover, subjects with successful weight loss are used to experiencing weight regain within 1 year, and approximately 20–35% of men are reported to experience weight fluctuation repeatedly^6,7^.

Recently, repeated weight fluctuation and weight cycling have been documented as risk factors for increasing morbidity and mortality, including cancer^8^. Since the 1990s, several studies have reported that weight changes affect an increased cancer incidence, such as renal cell cancer^9^, and obesity-related cancers, including breast cancer, endometrial cancer, and colorectal cancer^5,10–13^.

According to the World Cancer Report released by the World Health Organization (WHO) in 2018, cancer is the second leading cause of mortality worldwide and was responsible for an estimated 9.6 million deaths in 2018 and globally approximately 1 in 6 deaths (Fact sheets on cancer, WHO). According to the Korean Statistical Information Service (KOSIS) in 2017, the number of deaths from cancer was 154 per 100,000 persons, which is the leading cause of mortality in Korea, and one in three men and one in four women have suffered from cancer at least once in their lifetime. In Korea, a few studies have demonstrated that frequent weight change affects the development of papillary thyroid cancer^14,15^.

Smoking is one of the most important risk factors for cancer development and is responsible for approximately 22% of cancers^16^. In addition, smoking habits affect weight and BWV^17–19^.

Therefore, the aim of this study was to assess the association between BWV and cancer incidence and site-specific cancer incidence and to assess the extent to which the association varies with smoking status in Korean men using data from a retrospective population-based longitudinal study.

## Results

During 7,015,413 person-years of follow-up, 11,494 men (0.65%) developed new-onset cancers. The baseline characteristics of the study population according to the quintile groups of BWV are presented in Table 1. The mean weight variability values of the ASV quintile groups were 0.90 kg, 1.39 kg, 1.74 kg, 2.21 kg, and 3.13 kg. As the BWV quintile increased, initial weight, mean weight and initial BMI increased, and the proportion of the subjects with weight gain or weight loss was greatest in the highest quintile BWV group (Table 1).

**Table 1:** Baseline characteristics of the study population according to body weight variability.

	Quintile 1 (n = 357,687)	Quintile 2 (n = 341,665)	Quintile 3 (n = 290,578)	Quintile 4 (n = 433,705)	Quintile 5 (n = 336,213)	p-value
Range of ASV (kg)	≤ 1.22	1.22–1.56	1.56–1.89	1.89–2.5	> 2.5	
Mean ASV (kg)	0.90 $\pm$ 0.24	1.39 $\pm$ 0.11	1.74 $\pm$ 0.09	2.21 $\pm$ 0.19	3.13 $\pm$ 0.41	< 0.0001
Age (years)	49.43 $\pm$ 7.57	49.45 $\pm$ 7.84	49.03 $\pm$ 7.47	49.93 $\pm$ 8.11	50.34 $\pm$ 8.41	< 0.0001
Initial weight (kg)	66.20 $\pm$ 8.43	67.00 $\pm$ 8.56	67.67 $\pm$ 8.70	68.28 $\pm$ 9.06	69.43 $\pm$ 9.76	< 0.0001
Final weight (kg)	66.19 $\pm$ 8.57	66.93 $\pm$ 8.73	67.59 $\pm$ 8.90	68.07 $\pm$ 9.26	68.99 $\pm$ 9.94	< 0.0001
Mean weight (kg)	66.21 $\pm$ 8.40	67.00 $\pm$ 8.48	67.67 $\pm$ 8.57	68.23 $\pm$ 8.86	69.27 $\pm$ 9.38	< 0.0001
Initial BMI (kg/m^2^)	23.61 $\pm$ 2.55	23.82 $\pm$ 2.59	23.97 $\pm$ 2.63	24.14 $\pm$ 2.73	24.44 $\pm$ 2.93	< 0.0001
Initial BMI (BMI ≥ 25) (n, %)	107,084 (29.94)	112,462 (32.92)	102,177 (35.16)	164,424 (37.91)	142,811 (42.48)	< 0.0001
Initial BMI (BMI ≥ 30) (n, %)	2940 (0.82)	3747 (1.10)	4148 (1.43)	8540 (1.97)	11,452 (3.41)	< 0.0001
Final BMI (BMI ≥ 25) (n, %)	111,361 (31.13)	115,363 (33.76)	103,880 (35.75)	163,893 (37.79)	138,634 (41.23)	< 0.0001
Final BMI (BMI ≥ 30) (n, %)	3532 (0.99)	4558 (1.33)	4979 (1.71)	9942 (2.29)	12,051 (3.58)	< 0.0001
**Weight change (n, %)**^**a**^						< 0.0001
Stable	289,317 (80.89)	234,688 (68.69)	186,542 (16.56)	247,195 (21.94)	168,717 (14.98)	
Gain	35,015 (9.79)	52,891 (15.48)	51,394 (17.69)	87,991 (20.29)	75,841 (22.56)	
loss	33,355 (9.33)	54,086 (15.83)	52,642 (18.12)	98,519 (22.72)	91,655 (27.26)	
Fasting plasma glucose (mg/dl)	96.64 $\pm$ 25.49	97.17 $\pm$ 27.16	97.51 $\pm$ 27.97	98.31 $\pm$ 29.73	99.78 $\pm$ 32.82	< 0.0001
Total cholesterol (mg/dl)	197.96 $\pm$ 35.90	198.40 $\pm$ 36.33	198.67 $\pm$ 36.36	198.89 $\pm$ 36.80	199.22 $\pm$ 37.50	< 0.0001
**Smoking status (n, %)**						< 0.0001
Non-smoker	152,986 (42.77)	144,476 (42.29)	116,311 (40.03)	175,110 (40.38)	131,845 (39.21)	
Ex-smoker	64,600 (18.06)	61,187 (17.91)	50,676 (17.44)	74,536 (17.19)	55,897 (16.63)	
Current-smoker	140,101 (39.17)	13,602 (39.81)	123,591 (42.53)	184,059 (42.44)	148,471 (44.16)	
**Alcohol consumption**^**b**^						< 0.0001
Non	115,434 (32.27)	112,103 (32.81)	94,357 (32.47)	144,715 (33.37)	114,930 (34.18)	
Low risk	213,598 (59.72)	200,190 (58.59)	171,835 (59.14)	247,693 (57.11)	186,835 (55.57)	
Moderate risk	12,732 (3.56)	12,962 (3.79)	10,457 (3.60)	14,254 (3.98)	13,808 (4.11)	
High risk	15,923 (4.45)	14,610 (4.80)	13,929 (4.79)	24,043 (5.54)	20,640 (6.14)	
**Physical activity (n, %)**						< 0.0001
Low (0 day)	155,714 (43.53)	152,351 (44.59)	130,706 (44.98)	201,697 (46.51)	162,256 (48.26)	
Moderate (1–4 days)	170,861 (47.77)	158,235 (46.31)	135,371 (46.59)	192,282 (44.33)	142,843 (42.49)	
High (5–7 days)	31,112 (8.70)	31,079 (9.10)	24,501 (8.43)	39,726 (9.16)	31,114 (9.25)	
Hypertension (n, %)^c^	129,275 (36.14)	128,204 (37.52)	111,462 (38.36)	171,739 (39.60)	140,057 (41.66)	< 0.0001
Diabetes Mellitus (n, %)^d^	30,387 (8.50)	31,956 (9.35)	27,616 (9.50)	45,216 (10.43)	39,526 (11.76)	< 0.0001
Dyslipidemia(n, %)^e^	44,537 (12.45)	13,890 (12.85)	37,577 (12.93)	57,954 (13.36)	46,398 (13.80)	< 0.0001

*ASV* average successive variability, *BMI* Body Mass Index.

^a^If the last weight was changed by more than 5% from the initial weight, it was defined as weight gain or loss, whereas we defined the weight as stable if the last weight was changed by less than 5% from the initial weight.

^b^Low, moderate and high risk alcohol consumption was defined as drinking less than 40 g per day, as drinking 40 to 60 g per day, and as drinking more than 60 g per day, respectively.

^c^Hypertension was defined as a systolic or diastolic blood pressure (BP) of ≥ 140/90 mm Hg or the use of antihypertensive medication or history of hypertension.

^d^Diabetes mellitus was defined as a fasting blood glucose ≥ 126 mg/dl or the use of antidiabetic medication or history of diabetes mellitus.

^e^Dyslipidemia was defined as a total cholesterol level ≥ 240 mg/dl or the use of statins.

Participants who had higher weight variability had higher fasting plasma glucose and total cholesterol, and the proportion of the subjects with hypertension, diabetes mellitus, and dyslipidemia also increased as the BWV quintile increased (36.14% and 41.66%, 8.50% and 11.76%, 12.45% and 13.80% for the lowest quintile (Q1) and the highest quintile (Q5), respectively). Current smokers and low-risk alcohol consumers were more likely to have greater weight variability, and those with moderate physical activity were more likely to have lower weight variability (Table 1).

### Associations between body weight variability and all cancers and site-specific cancers

We checked PH assumption with a log negative log plot, and p-values were > 0.1. In unadjusted models and minimally adjusted model (adjusted for age only or adjusted for age and mean weight), the highest BWV group had a statistically significant higher risk of all cancers compared to the lowest quintile group (Supplementary 1). Table 2 presents the HRs for all cancers according to the BWV. The highest BWV group had a statistically significant higher risk of all cancers compared to the lowest quintile group (HR 1.22, 95% CI 1.15–1.30). The total incidence of lung cancer was 176 per 100,000 persons (21.6% of all cancers), which was the most common, followed by stomach cancer, prostate cancer, colorectal cancer, and liver cancer (128 (15.7%), 103 (12.7%), 100 (12.3%), and 72 (8.8%), respectively). The risks for liver cancer, lung cancer and prostate cancer in the highest BWV quintile were significantly greater than the risks in the lowest quintile (HR 1.46, 95% CI 1.19–1.81 in Q5; HR 1.22, 95% CI 1.07–1.39 in Q5; and HR 1.36, 95% CI 1.15–1.62 in Q5, respectively).

**Table 2:** Hazard ratios for all cancer and site-specific cancers according to the quintile groups of body weight variability.

	Quintile 1	Quintile 2	Quintile 3	Quintile 4	Quintile 5
**All cancer**					
Events	1819	2091	1663	3050	2871
Person-years	1,426,925	1,362,335	1,158,869	1,728,475	1,338,808
Incident density^a^	127	153	144	176	214
HR	1.00 (ref)	1.07 (1.01–1.14)	1.09 (1.02–1.17)	1.12 (1.06–1.19)	1.22 (1.15–1.30)
**Colorectal**					
Events	238	275	203	350	332
Incident density^a^	17	20	18	20	25
HR	1.00 (ref)	1.08 (0.91–1.29)	1.04 (0.86–1.25)	0.99 (0.84–1.18)	1.10 (0.92–1.31)
**Kidney**					
Events	36	46	38	64	66
Incident density^a^	3	3	3	4	5
HR	1.00 (ref)	1.20 (0.77–1.86)	1.22 (0.77–1.93)	1.18 (0.78–1.78)	1.38 (0.91–2.10)
**Liver**					
Events	139	180	150	254	275
Incident density^a^	10	13	13	15	21
HR	1.00 (ref)	1.19 (0.96–1.49)	1.24 (0.98–1.56)	1.18 (0.96–1.46)	1.46 (1.19–1.81)
**Lung**					
Events	381	429	364	696	623
Incident density^a^	27	31	31	40	47
HR	1.00 (ref)	1.04 (0.90–1.19)	1.13 (0.98–1.31)	1.19 (1.05–1.35)	1.22 (1.07–1.39)
**Prostate**					
Events	217	285	198	403	365
Incident density^a^	15	21	17	23	27
HR	1.00 (ref)	1.24 (1.04–1.48)	1.13 (0.94–1.38)	1.28 (1.08–1.52)	1.36 (1.15–1.62)
**Stomach**					
Events	314	322	257	464	445
Incident density^a^	22	24	22	27	33
HR	1.00 (ref)	0.97 (0.83–1.13)	0.99 (0.84–1.17)	1.01 (0.87–1.16)	1.12 (0.97–1.31)
**Other**					
Events	517	579	473	854	797
Incident density^a^	36	43	41	49	60
HR	1.00 (ref)	1.04 (0.93–1.18)	1.08 (0.95–1.23)	1.1 (0.99–1.23)	1.20 (1.07–1.34)

Model was adjusted for age, hypertension, diabetes mellitus, dyslipidemia, moderate physical activity, alcohol consumption, current smoker, mean weight and weight change.

^a^Incident density is calculated as cases divided by 100,000 person-years.

### The association between body weight variability and the risk of all cancers and site-specific cancers according to subgroups

**Figure 1:**
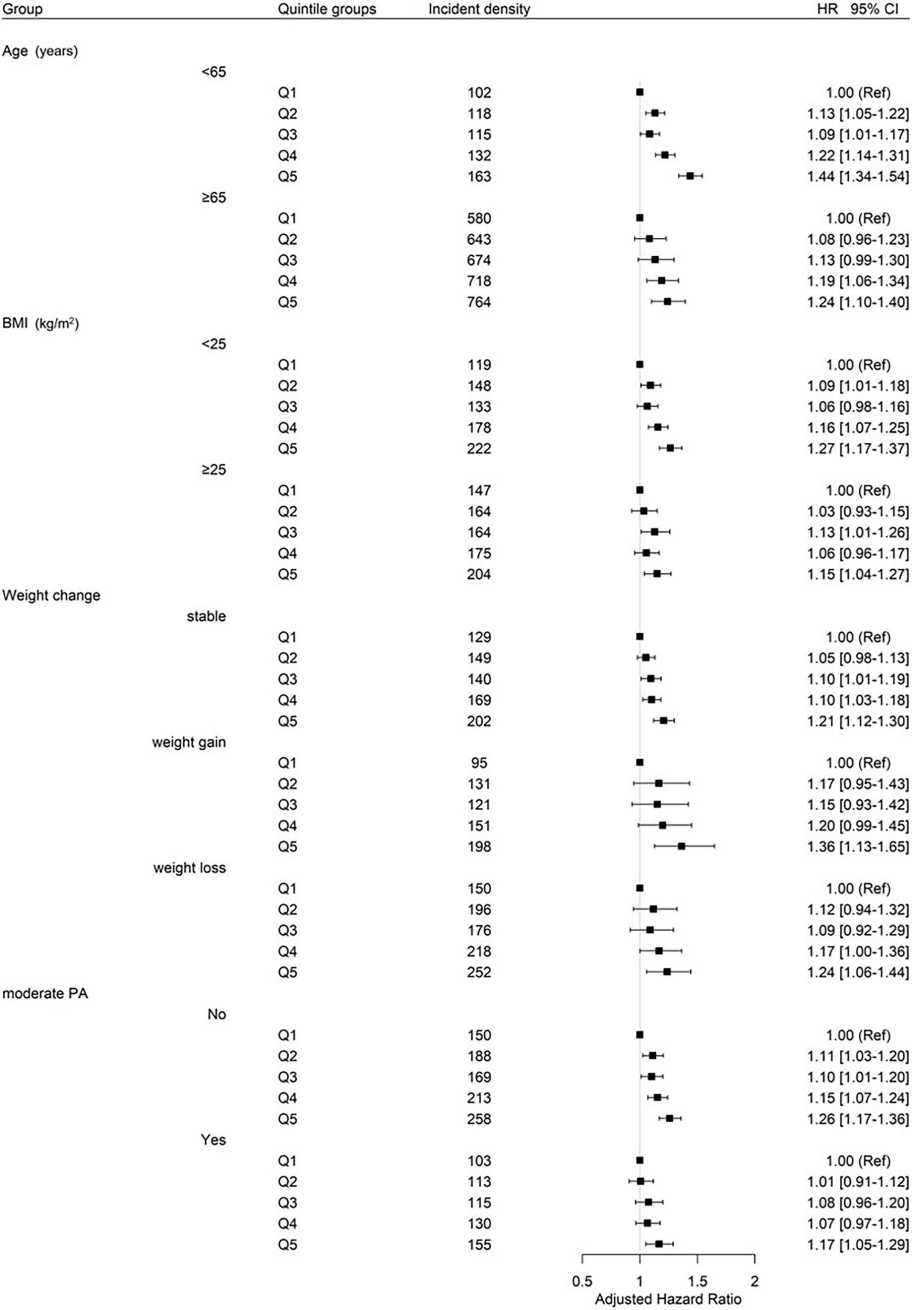
Stratified analyses of the effect of body weight variability on all cancers according to subgroups of age, initial body mass index, weight change and physical activity.

To evaluate the effect of BWV on the all cancer incidence according to subgroups of age, initial BMI, weight change and moderate physical activity, stratified analyses were conducted (Fig. 1). Participants with the highest BWV quintile compared to the lowest quintile had a significantly elevated risk of all cancers regardless of age group (< 65 years and ≥ 65 years) (HR 1.44, 95% CI 1.34–1.54 and HR 1.24, 95% CI 1.10–1.40, respectively). When the subjects were divided according to initial BMI, the risk of all cancers was increased in the initially normal weight group (BMI < 25 kg/m^2^) as well as in the obese group (BMI ≥ 25 kg/m^2^) according to the increase in BWV quintiles, and the risk was the greatest in the highest BWV quintile normal weight group (HR 1.27, 95% CI 1.17–1.37; HR 1.15, 95% CI 1.04–1.27, in Q5). We conducted stratified analysis to check the differential effect of BWV on the risk of all cancer and site-specific cancers by obesity status, stronger evidences were observed in the non-obese individuals. Evidence for all cancer was weaker in obese group and the associations between BWV and the risk of site-specific cancers were no longer statistically significant (Supplementary 2). In the initially non-obese weight loss group, a 150% increased risk of liver cancer was observed with increasing BWV. However, the risk of prostate and lung cancer in the highest quintile of BWV compared to the lowest significantly increased only in the initially non-obese and weight stable group (Supplementary 2). The risk of all cancers was increased among those with the highest BWV compared to those with the lowest BWV for all subgroups of weight change during the follow-up period; those who had stable weight (HR 1.21, 95% CI 1.12–1.30), those who gained weight (HR 1.36, 95% CI 1.13–1.65), and those who lost weight (HR 1.24, 95% CI 1.06–1.44). All participants with the greatest BWV had the highest risk of cancer incidence regardless of physical activity status.

**Figure 2:**
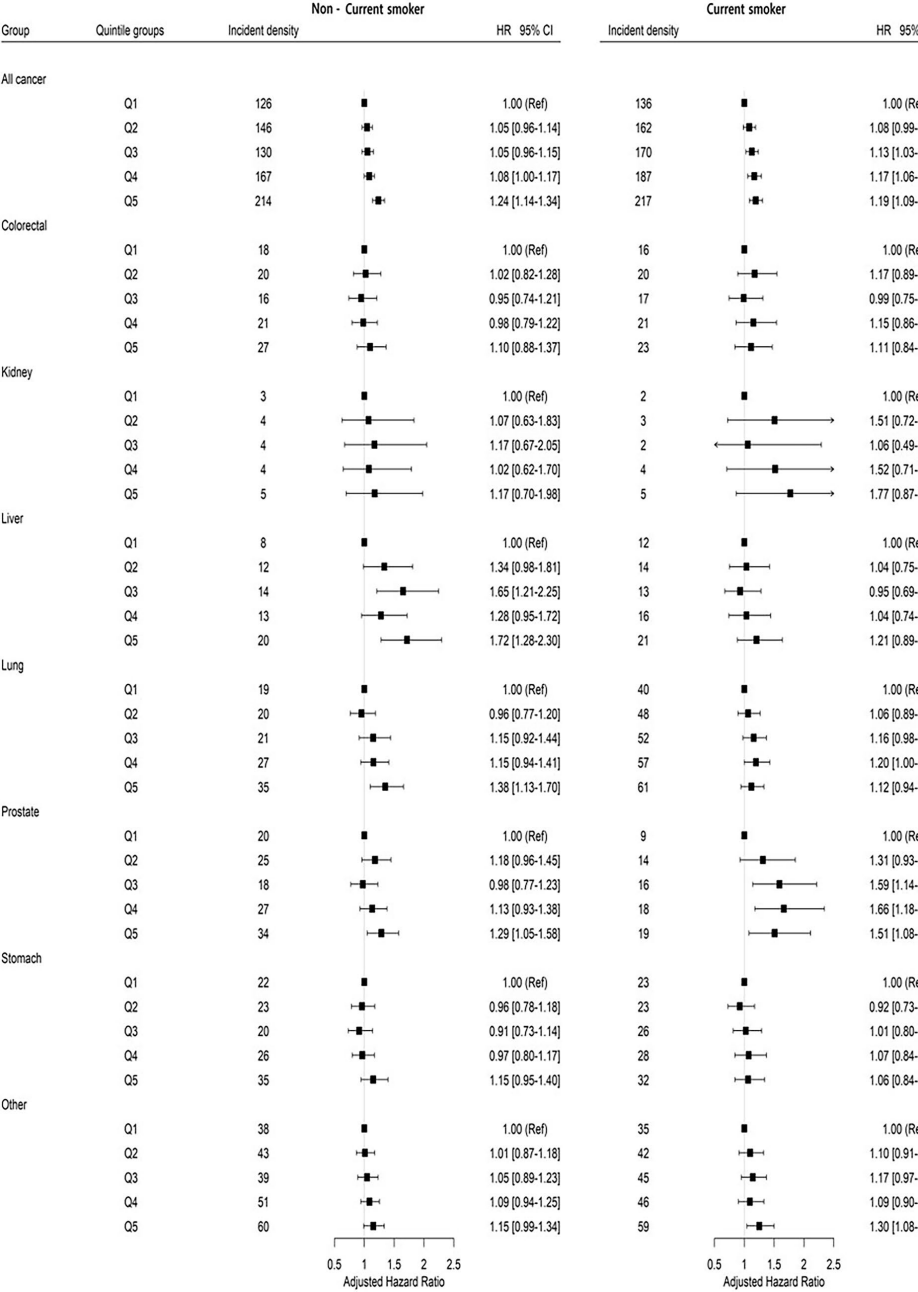
The association between body weight variability and the risk of all cancers and site-specific cancers according to smoking status (current smokers vs non-current smokers).

The risk for all cancers according to BWV stratified by smoking status is shown in Fig. 2. Participants with the highest BWV of the noncurrent smokers had a greater risk of all cancers compared to those with the lowest BWV (HR 1.24, 95% CI 1.14–1.34). The risks of liver cancer, lung cancer and prostate cancer showed the statistically significant highest hazard ratio among those with the highest BWV compared to those with the lowest (HR 1.72, 95% CI 1.28–2.30; HR 1.38, 95% CI 1.13–1.70; and HR 1.29 95% CI 1.05–1.58 in Q5 vs Q1, respectively). Similarly, the risk of all cancers in current smokers was also increased with an increase in the BWV quintile (HR 1.19, 95% CI 1.09–1.31 in Q5). The HRs of the individuals with the highest BWV were significantly higher than those with the lowest BWV in prostate cancer and other cancers (HR 1.51, 95% CI 1.06–2.11 and HR 1.30, 95% CI 1.08–1.55, respectively) but not in liver and lung cancer.

### Sensitivity analyses of the association between body weight variability and all cancer

Sensitivity analyses were conducted by adjusting for initial BMI or initial weight instead of the mean weight and showed similar results.

## Discussion

In this retrospective population-based longitudinal study, we found that strong evidence of the effect of BWV on the risk of cancer incidence in men, in lung, liver, and prostate cancer. Evidence was weaker and it was no longer statistically significant in kidney cancer. A positive association between BWV and the risk of total cancer was observed regardless of weight change (weight stable, weight gain, weight loss) and obesity status (obese or nonobese, BMI < 25 kg/m^2^ or BMI ≥ 25 kg/m^2^). The positive associations between BWV and total and site-specific cancer incidences, such as liver, lung, and prostate cancer, were still significant in noncurrent smokers; however, in current smokers, the effect size of BWV on cancer risk reduced statistically in lung and liver cancer but remained high in prostate cancer.

Many studies have been conducted on weight and cancer risk or weight gain and obesity-related cancer risk, whereas several studies have examined the association between BWV and cancer^11,13,20–24^. Most previous studies showing a positive association between weight cycling or fluctuation and cancer risk were conducted in women^11,13,20–26^, especially in obesity-related cancers such as breast cancer, endometrial cancer, and renal cancer^25,26^. Meanwhile, a few studies in men showed a null association between weight change or cycling and cancer incidence, including prostate cancer^5,27^.

One study demonstrated null associations between weight cycling and 15 cancer incidences in a large prospective cohort of 132,312 men and women, independent of BMI^5^. The investigators retrospectively collected data for weight cycling by self-reported intentional weight loss and regaining (more than 10 lbs.) episodes throughout the subjects’ lifetime. We also analyzed the BWV-related risk of prevalent cancers in Korean men and found the risk of all cancers to be significantly increased with increasing BWV with a dose–response relationship. A positive relationship between BWV and the risk of total cancer was observed in all age group and obesity status, and all direction of weight change, and people who did moderate physical activity.

Most prior studies have shown a relationship between weight cycling or fluctuation and obesity-related cancers^9,26,28^, especially among subjects who gained weight during the study period^8,20,29^. However, our study population is mainly composed of initially not severely obese men (initial BMI ≥ 25 kg/m^2^ (35.7%), initial BMI ≥ 30 kg/m^2^ (1.75%)) by Asia–Pacific obesity criteria^30^, even nonobese men based on the obesity criteria according to the WHO definition (https://www.who.int/news-room/fact-sheets/detail/obesity-and-overweight). Moreover, most of our study population (72.25%) belonged to the weight stable or weight loss group during the follow-up period. A positive relationship between BWV and the risk of total cancer was observed in nonobese and obese individuals. Therefore, the impact of obesity (measured by BMI) itself on BWV-related cancer risk might be much smaller than those found in previous Western studies. Among the top 5 prevalent cancers in men aged over 60 years, typical obesity-related cancers in Western countries, such as colorectal cancer and stomach cancer^29,31,32^, were not included in BWV-related cancer in this study.

In line with previous studies, a possible explanation for the association between BWV and cancer risk in mostly nonobese men might be as follows. Weight fluctuation is related to loss of lean muscle mass and weight regain with increased adiposity, especially in the aging population^13,33–37^. Low muscle mass as well as increased adiposity even in nonobese men results in an altered metabolic^38^ and immune response by inducing changes in natural killer cell cytotoxicity^39,40^. Weight cycling with increased T-cell accumulation and enhanced inflammatory response ultimately leads to chronic inflammation and might decrease immune surveillance for abnormally transformed cells and increase the risk of cancer development^40–42^.

According to race, age and sex, the common site-specific cancers are quite different. The top five prevalent cancers in Korean men aged over 60 years are stomach, lung, prostate, colorectal, and liver cancer, while those in American men are prostate, lung, colorectal, renal cancer, and malignant melanoma (National Cancer Information Center 2017).

Among the prevalent cancers in Korean men, our results showed a positive relationship between BWV and site-specific cancer, especially liver, lung and prostate cancer, independent of initial BMI or mean body weight.

In 2018, primary liver cancer (PLC) was the 5th most common cancer among men worldwide (International Agency for Research on Cancer. Liver. World Health Organization. Available at http://gco.iarc.fr/today/data/factsheets/cancers/11-Liver-fact-sheet.pdf. 2018; Accessed: March 2, 2020), and the 4th most common malignancy in Korean men^43^.

Hepatocellular cancer (HCC), which represents approximately 85% of PLC in Korea, develops mainly within a background of advanced liver disease related to alcoholic and viral hepatitis B and C^44^. Recently, obesity has emerged as a newly established risk factor for HCC worldwide, and the risk of HCC is increased up to two times in obese individuals compared to normal weight individuals^45^.

In this study, the risk of PLC increased by 46% in the highest BWV quintile group, which is the highest increase among all cancers. The mean initial BMI in the subjects with PLC was 24.8 kg/m^2^, and the proportion of initially obese subjects (BMI ≥ 25 kg/m^2^) in the highest BWV quintile group was 42.5% and increased with increasing BWV.

The individuals in the highest BWV quintile group were more likely to be obese and physically inactive and less likely to be weight stable. When the total subjects were divided into weight stable, weight gain, and weight loss groups by a weight change of 5% or more of initial body weight, the risk of PLC in the highest BWV quintile group compared to the lowest quintile significantly increased in initially nonobese subjects with weight loss, independent of initial BMI and weight change. A statistically insignificant similar association was observed in the initially obese and weight gain groups because of the low incidence of PLC (0 cases of PLC in the lowest BWV quintile group). The aHR (95% CI) of PLC in the highest quintile group compared to the 2nd quintile in the initially obese and weight gain groups was 1.70 (0.63–4.59). This result is in line with a previous study showing the different impact of a BMI increase on the risk of liver cancer in underweight and obese men (J-shaped association between BMI and the risk of liver cancer)^46^.

In the initially obese weight gain group, weight fluctuation associated with increased adiposity, followed by insulin resistance and hepatic steatosis, ultimately might lead to hepatic carcinogenesis by oxidative stress, lipotoxicity and stimulation of the IGF-1 axis by hyperinsulinemia^47^. Repeated exposure to high leptin and low adiponectin due to increased adiposity could be involved in NAFLD progression, hepatic fibrosis and the altered immune response and angiogenesis, which eventually result in hepatic carcinogenesis^48^. On the other hand, in the initially nonobese weight loss group, a 150% increased risk of liver cancer was observed with increasing BWV (Supplementary 2).

Oxidative stress and chronic inflammation are considered critical pathophysiological mechanisms in cancer^49^. In this study, subjects in the highest BWV quintile group were more likely to be older, current smokers, and moderate or heavy drinkers, who might have sustained oxidative stress, chronic inflammation and more pathologic vasculature^49,50^. Older, nonobese, weight variable individuals with weight loss often suffer from protein energy undernutrition and a lack of functional reserve to cope with chronic oxidative stress and inflammation, which in turn lead to hepatic carcinogenesis. Furthermore, research to make an inference on the underlying mechanism of these associations is needed.

Prostate and lung cancer are the 1st and 2nd most common cancers in men worldwide based on the 2018 WHO and IARC report^51^ and the 4th and 2nd most common malignancies in Korean men^52^.

In stratified analyses according to obesity status and weight change, the risk of prostate and lung cancer in the highest quintile of BWV compared to the lowest significantly increased only in the initially nonobese and weight stable group (Supplementary 2).

Obesity is considered a predictor of prostate cancer mortality and recurrence^53^. However, the association between obesity and the risk of prostate cancer is still controversial. Moreover, a previous study showed an inverse association between BMI and lung cancer^46,54^.

The underlying mechanism of weight variability and cancer in nonobese subjects might be as follows: a period time of weight fluctuation may have profound inflammatory consequences on the body compared to a similar period of sustained weight gain in mice^55^. Rapid weight regain will cause a greater amount of cell stress due to rapid fat cell hypertrophy, leading to excessive cytokine release^56^, even in nonobese individuals.

Therefore, weight variability-related rapid fat cell hypertrophy may induce altered angiogenesis to keep up with the rapidly expanding fat mass, resulting in hypoxia and necrosis and inflammation to increase the risk of cancer development.

We further conducted stratified analyses to investigate whether the associations between BWV and total and site-specific cancers originated from the effect of smoking. The results were different when dividing the entire group of subjects into current smokers and noncurrent smokers.

The associations between BWV and total and site-specific cancers were similar to those of the total study population in noncurrent smokers, whereas in current smokers, the effect of BWV on cancer incidence in the liver and lung was lower in current smokers than non-smokers and was not statistically significant. The association remained strong and statistically significant in prostate cancer.

PLC and lung cancer are the most representative smoking-related cancers classified by the CDC (https://www.cdc.gov/cancer/tobacco/). Generally, smoking is considered a much stronger risk factor for cancer than excess body weight^57^. In a previous study, the risks of lung and liver cancer in heavy smokers who smoked over 1 pack of cigarettes per day increased up to 9.8 and 3.4, respectively, and those in light smokers were 3.1 and 1.7, respectively, compared to those in nonsmokers^57^. The HRs of liver cancer in the overweight (25 ≦ BMI < 30 kg/m^2^) and obese (BMI ≥ 30 kg/m^2^) groups increased up to 1.28 (0.77–2.12) and 2.21 (1.14–4.01), respectively. Therefore, the effect of BWV on the increased risk of cancer, at most approximately 50%, cannot compromise the effect of smoking on smoking-related cancers such as lung and liver cancer.

Another explanation might be confounding by the amount of smoking. Higher BWV-associated cancer risk, especially in nonobese individuals, might be a proxy for heavy smoking when we categorized smoking crudely as current smokers, ex-smokers, and nonsmokers.

Meanwhile, prostate cancer was neither an obesity-related cancer nor a smoking-related cancer as classified by the CDC (https://www.cdc.gov/cancer/). We found that in current smokers, only prostate cancer significantly increased with increasing BWV. A higher cumulative exposure to cigarette smoking in current smokers, but not in ex-smokers, was reported to be related to significantly higher cerebral blood flow levels in the occipital and temporal lobes and in deep cortex structures compared to levels in nonsmokers^58^.

Recent research also showed heterogeneous associations between smoking and a wide range of lifetime risks of cardiovascular diseases in 12,937,380 people in England^59^. Remarkable differences in the strength of associations were reported across various cardiovascular endpoints, ranging from weak with stable angina (adjusted HR (aHR) 1.08, 95% CI 1.01–1.15) and very strong with abdominal aneurysm and peripheral artery disease (range 2.70 to 5.18). They reported that the disease with the highest lifetime risk in current smokers was peripheral artery disease. In our study, the risk of prostate cancer increased with increasing BWV, and the relationship was even reinforced in current smokers. Smoking-related redistribution of blood flow to brain structures and severe atherosclerotic vascular changes in the lower part of the body might increase the possibility of the increased risk of BWV-related cancer development in the prostate, especially in current smokers, but not in the lung or liver. Further investigation is needed to validate our results.

This is the first study showing the positive association between BWV and cancer incidence in men and the differential effect of smoking status on the association between BWV and site-specific cancers such as lung, liver and prostate cancer.

The most important strength of this study is that it is a large-scale longitudinal study involving 1,759,848 subjects in their mid to older ages that examined nationally representative, reliable NHIS-HEALS data. Second, we obtained data using the actual measured weights, not the self-reported weight changes of subjects. Finally, only first-time cancer diagnosis was assessed to ensure that previous cancer did not influence future cancers.

However, there are several limitations in our study. First, BWV was not estimated after the final weight measurement until 2015, and possible weight changes after the index date and between health examinations were not accounted for. Seconds, based on the retrospective nature of the study design, we could not measure the exact changes in fat mass or lean mass with BWV, and the intentionality of weight fluctuation could not be assessed in our study. Third, since we restrict cancer definitions to men who were hospitalized for more than 2 days, there might be a possibility of excluding early cancer in situ such as colon cancer diagnosed and removed by colonoscopy. Fourth, because taking medical checkups for enrolled in the NHIS is not mandatory, the participation rate ranged from 48.0 to 72.9% between the year 2003 and 2012. Our study population was composed of those who participated more than 5 times of health examinations. We could not conduct a sensitivity analysis which included subjects who underwent less than 5 health examinations to address the possibility of selection bias. Residual or unmeasured confounding such as socio-economic status and other health related reasons for BWV still existed. In addition, the possibility of protopathic bias where BWV is a symptom of undiagnosed cancer still remained. Weight loss is common among people with cancer. According to the American Cancer Society, unexplained weight loss is often the first noticeable symptom of cancers of the esophagus, pancreas, stomach, and lung. Although we excluded men who were diagnosed with cancer between 2002 and 2011 (n = 13,861), it is possible to include undiagnosed cancer patients especially in weight loss group.

Finally, we assumed a log-linear association between the risk of cancer incidence and the continuous confounders such as age and mean weight. If there is a non-linear relationship, treating these confounders as a linear predictor, might result in an deflated assessment of the statistical significance of the association between one of the variables and the risk of cancer incidence and residual confounding might be remained.

Further studies on the effect of fat mass and lean mass variability, including intentionality of weight change, and the possible cause of BWV on health outcomes are needed.

In this nationally representative, population-based cohort study, we showed BWV as a predictor of cancer incidence in men and the differential effect of smoking on the association between BWV and site-specific cancers such as lung, liver and prostate cancer. It is necessary to initiate longitudinal studies that combine instrumental properties to measure body composition, and its pathophysiological mechanisms.

## Methods

### Data collection

We used data from a nationwide population-based database of the National Health Insurance Service (NHIS) in Korea^60^. The NHIS system is an obligatory health insurance program that offers universal medical care coverage to the entire Korean Population (approximately 50 million people). Enrollee in the NHIS are recommended to receive a standardized medical health checkups at least every 2 years.

The NHIS database includes information on subjects’ demographics, health behaviors and laboratory tests. Self-reported questionnaires for health behaviors such as alcohol consumption, smoking status and regular exercise and for past medical histories such as cardiovascular disease (CVD) and cancer and family history of CVD are also included. The overall participation rate is 76.1% of the eligible population in 20 (https://www.mohw.go.kr). This study was conducted according to the Declaration of Helsinki. The Institutional Review Boards (IRBs) of Seoul National University Hospital (IRB number: 1911-031-1077) approved this study and waived the need for informed consent because the NHIS database used in this study was anonymized in accordance with South Korean personal data protection laws.

### Study population

**Figure 3:**
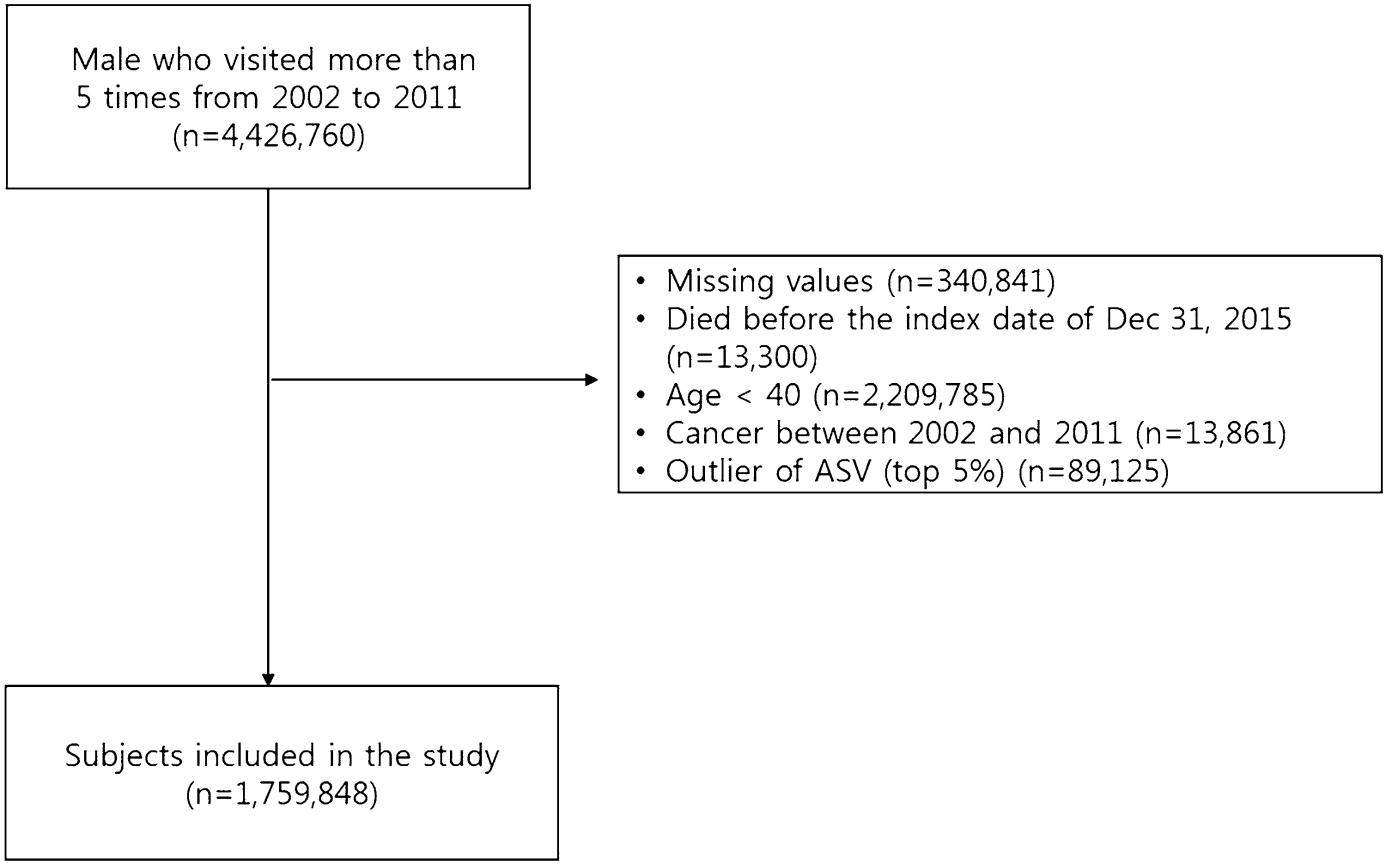
Flow diagram of the study population.

Data for 4,426,790 male subjects who underwent health examinations more than 5 times from 2002 to 2011 were used. The following were excluded: (1) participants with missing important covariates (n = 340,841); (2) those who died before the index date of December 31, 2015; (3) those who had outliers of ASV (top 5%, n = 89,125). Finally, 1,759,848 male subjects were included in this study (Fig. 3). All subjects were followed-up from January 1, 2012, until date on which they were diagnosed with cancer or December 31, 2015, whichever came first.

### Definition of weight variability and covariates

Variability in weight was defined as the average of the absolute difference between successive values (ASV), which is one of the methods to define intra-individual variability in weight between visits for health examination^61^. We divided subjects into 5 categories according to the quintiles of weight variability: ASV less than 1.22 kg (Q1); 1.22 kg to less than 1.56 kg (Q2); 1.56 kg to less than 1.89 kg (Q3); 1.89 kg to less than 2.5 kg (Q4); and 2.5 kg or greater (Q5).

Detailed definition of all covariates included in the analyses are presented in Supplementary Table S4. Hypertension (HTN) was defined as a systolic or diastolic blood pressure (BP) of ≥ 140/90 mm Hg or the use of antihypertensive medication or history of hypertension. Diabetes mellitus was defined as a fasting blood glucose ≥ 126 mg/dl or the use of antidiabetic medication or history of diabetes mellitus. Dyslipidemia was defined as a total cholesterol level ≥ 240 mg/dl or the use of statins based on the guideline for statin use in Korea (Supplementary 3).

Low physical activity was defined as 0 days of exercise per week, moderate physical activity was determined as 1 to 4 days of exercise per week and high physical activity was specified as 5 to 7 days of exercise per week regardless of the intensity of physical activity^62^. Low-, moderate- and high-risk alcohol consumption were defined as drinking less than 40 g per day, 40 to 60 g per day, and more than 60 g per day, respectively. If the last weight was changed by more than 5% from the initial weight, it was defined as weight gain or loss, whereas we defined the weight as stable if the last weight was changed by less than 5% from the initial weight.

Cancer was defined when the subject had a cancer code (C00–C97), by the Tenth Revision of the International Classification of Diseases (ICD-10) codes, and was hospitalized for more than two days. We further categorized cancer as colorectal cancer (C18–C20), kidney cancer (C46), liver cancer (C22), lung cancer (C33–C34), prostate cancer (C61), stomach cancer (C16), and other cancers (other codes starting with C).

### Statistical analysis

Numerical variables are represented as the means ± standard deviations (SD), and categorical variables are expressed as frequencies and percentages (%). The risk of cancer from BWV was identified using Cox proportional hazards regression analysis by estimating the hazard ratios (HRs) and 95% confidence intervals (CIs) after adjusting for age, mean weight, hypertension, diabetes mellitus, dyslipidemia, and health behaviors such as moderate physical activity, alcohol consumption, smoking status (nonsmoker, ex-smoker, current smoker), and weight change (stable, gain, loss). Subgroup analyses were also conducted according to age, initial BMI (or initial body weight or mean body weight), weight change (loss, stable, gain), and moderate physical activity. To evaluate the impact of smoking on total land site-specific cancer, a stratified analysis on smoking status (noncurrent smoker vs current smoker) was conducted. Sensitivity analyses were conducted adjusting for initial BMI or initial weight instead of the mean weight. Statistical significance was determined as p < 0.05 with two-sided tests. To test the proportional hazard(PH) assumption, cox.zph function of package “survival” from R software was used. This function creates Schoenfeld scaled residuals for each of the variables used in the model to test the individual PH assumption as well as the global PH assumption of the model. Statistical analysis was performed using SAS, version 7.1 (SAS Institute Inc.), and R software, version 3.5 (the R Foundation for Statistical Computing).

## Supplementary Information

Supplementary Information.
