# Supplementary material for: Body weight variability and cancer incidence in men aged 40 years and older-Korean National Insurance Service Cohort
Source: Sci Rep. 2021 Jun 9;11:12122. doi: 10.1038/s41598-021-91601-9 (PMC8190310; doi:10.1038/s41598-021-91601-9)
Supplement: Supplementary file 1 — Supplementary Information. [file 41598_2021_91601_MOESM1_ESM.docx]

**Supplementary Appendix**

**Supplementary 1. Hazard ratios(HR) and 95% confidence intervals (95% CI) for all cancer by body weight variability**

| Variable  ASV | Incidence Density | Unadjusted HR (95% CI) | Model 1^a^  Adjusted HR (95% CI), | Model 2 ^b^  Adjusted HR (95% CI), | Model 3 ^c^  Adjusted HR (95% CI), |
| --- | --- | --- | --- | --- | --- |
| Quintile 1 | 127 | 1 (reference) | 1(reference) | 1(reference) | 1(reference) |
| Quintile 2 | 153 | 1.09 (1.05-1.14) | 1.08(1.03-1.12) | 1.07 (1.03-1.11) | 1.07 (1.03- 1.11) |
| Quintile 3 | 144 | 1.13 (1.09-1.18) | 1.12(1.07-1.16) | 1.10 (1.06-1.14) | 1.10 (1.06-, 1.14) |
| Quintile 4 | 176 | 1.31(1.26-1.37) | 1.24 (1.19-1.29) | 1.21 (1.16-1.26) | 1.21 (1.16- 1.26) |
| Quintile 5 | 214 | 1.649(1.58-1.71) | 1.47 (1.41-1.53) | 1.42 (1.36-1.47) | 1.42 (1.36- 1.47) |

Abbreviations: ASV, Average Successive Variability of body weight;

Incident Density was calculated as cases divided by 100,000 person-years

^a^ Model was adjusted for age

^b^ Model was adjusted for age, mean weight

^c^ Model was adjusted for age, hypertension, diabetes mellitus, dyslipidemia, moderate physical activity, alcohol consumption, current smoker, mean weight and weight change.

**Supplementary.2 The association between body weight variability and the risk of all cancers and site specific cancers stratified by initial BMI and direction of body weight change.**


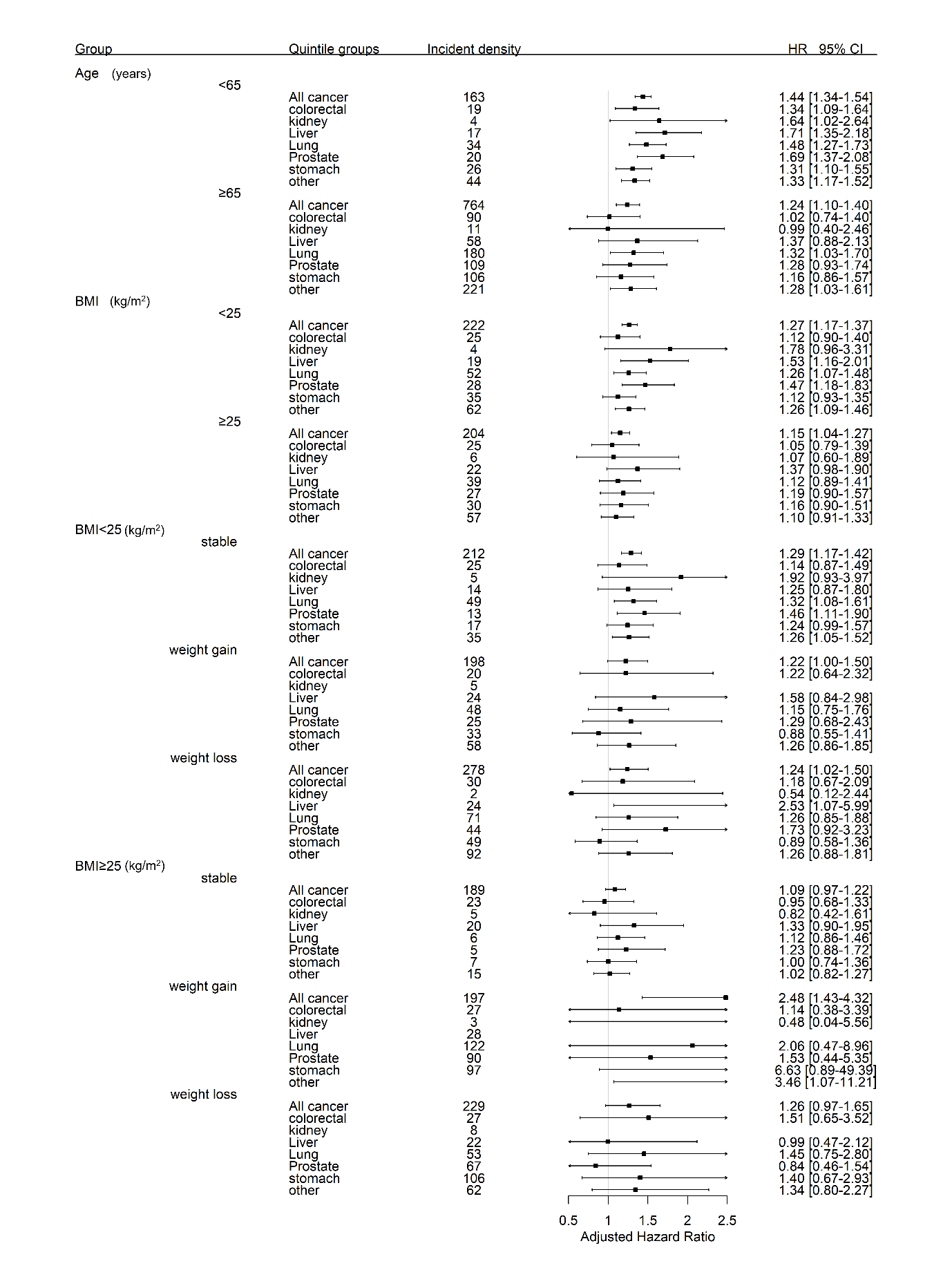


**Supplementary.3 Treatment guidelines for dyslipidemia in Korea**

* Risk classification and Target goal : LDL-C

1. Ultra-high risk group: coronary artery disease, ischemic stroke, transient ischemic attack, peripheral vascular disease

2. High risk group: carotid artery disease, abdominal aneurysm, diabetes

3. Major risk factors: smoking, high blood pressure, HDL-C <40mg/dL, men 45 years old or older, women 55 years old or older, family history of coronary artery disease early (parents, siblings men under 55 years old, women under 65 years old)

** For HDL-C 60mg/dL or more, one is subtracted from the total number of risk factors (total number of risk factors -1).

| **Risk classification** | **Target LDL-C (mg/dL)** |
| --- | --- |
| Ultra-high risk group | < 70 |
| High risk group | < 100 |
| Moderate risk group  ** Major risk factors ≥ 2 | < 130 |
| Low risk group  ** Major risk factors ≤ 1 | < 160 |

**Supplementary4. Definition of covariates using the subject's visit of health examination.**

| Variables |  |
| --- | --- |
| Mean ASV(kg) | All health assessments |
| Age (years) | First health assessment |
| Initial weight (kg) | First health assessment |
| Final weight (kg) | Last health assessment |
| Mean weight (kg) | All health assessment |
| Initial BMI (kg/m^2^) | First health assessment |
| Initial BMI (BMI≥25) (n, %) | First health assessment |
| Initial BMI (BMI≥30) (n, %) | First health assessment |
| Final BMI (BMI≥25) (n, %) | Last health assessment |
| Final BMI (BMI≥30) (n, %) | Last health assessment |
| Weight change (n, %) ^a^ | First and last health assessment |
| Stable |  |
| Gain |  |
| loss |  |
| Fasting plasma glucose (mg/dl) | First health assessment |
| Total cholesterol (mg/dl) | First health assessment |
| Smoking status (n, %) | First health assessment |
| Non-smoker |  |
| Ex-smoker |  |
| Current-smoker |  |
| Alcohol consumption ^b^ | First health assessment |
| Non |  |
| Low risk |  |
| Moderate risk |  |
| High risk |  |
| Physical Activity (n, %) | First health assessment |
| Low (0 day) |  |
| Moderate (1-4 days) |  |
| High (5-7 days) |  |
| Hypertension (n, %) ^c^ | First health assessment |
| Diabetes Mellitus (n, %) | First health assessment |
| Dyslipidemia(n, %) ^e^ | First health assessment |
